# Endometrioma With Hemorrhagic Ascites Mimicking Ovarian Malignancy: A Case Report

**DOI:** 10.7759/cureus.113351

**Published:** 2026-07-25

**Authors:** Iván L Rosso, Maria Paula Morales Ortigoza, Angela Duque Jimenez, Andrés F Marín, Wilmer Aponte

**Affiliations:** 1 Radiology, Universidad Nacional de Colombia, Bogotá, COL

**Keywords:** endometrioma, endometriosis, hysterectomy, ovarian neoplasms, tumor biomarker

## Abstract

Endometriosis is a highly prevalent and morbid condition in women of reproductive age, often leading to well-known complications such as infertility, intestinal adhesions, and severe chronic pelvic pain.

There is a particular and less frequent entity, even in patients with no prior history of pelvic pain or endometriosis, that presents as a pelvic mass associated with hemorrhagic ascites. This finding can be confused with a malignant ovarian neoplasm, a diagnostic error with unfavorable outcomes and a significant impact on patients' lives.

We present the case of a young woman with no prior history of pelvic pain who presented with a right adnexal mass, ascites, pleural effusion, and elevated tumor markers. She underwent a radical hysterectomy with a presumption of an ovarian neoplasm involving the peritoneum. Imaging findings included a right ovarian bilobed lesion with high T2 signal intensity, a shading sign, a fluid-fluid level, and thick walls. The final pathological diagnosis was endometriosis.

## Introduction

Endometriosis is an inflammatory pathology closely linked to estrogen, traditionally defined as the ectopic presence of endometrial glands and stroma, most frequently affecting the pelvic peritoneum, ovaries, and rectovaginal septum [1]. It is a highly prevalent condition that affects approximately 10% of women of reproductive age and is frequently associated with pelvic pain, infertility, and substantial impairment in quality of life [2]. The most frequent clinical manifestations are infertility and chronic pelvic pain, which, in some cases, show a poor response to available treatments [2]. Notably, symptom severity does not necessarily correlate with disease extent. Women with Stage I (limited number of lesions and few adhesions) according to the revised American Society for Reproductive Medicine (rASRM) classification may present with severe pain and infertility, whereas some women with Stage IV disease, characterized by extensive lesions, endometriomas, and dense adhesions, may remain asymptomatic [3].

The phenotypes of endometriosis are heterogeneous; the most frequent include superficial endometriosis, deep infiltrative endometriosis, and ovarian endometriosis, also known as endometrioma. The diagnosis of each of these subtypes varies significantly. In the case of superficial peritoneal lesions, no imaging modality possesses sufficient sensitivity or specificity for non-invasive diagnosis, and laparoscopic visualization remains the gold standard [4]. Conversely, for the diagnosis of endometrioma, transvaginal ultrasound (TVUS) and magnetic resonance imaging (MRI) demonstrate high performance, with sensitivities of 93% and 95% and specificities of 96% and 91%, respectively [5].

However, hemorrhagic ascites has been described as a rare manifestation of endometriosis; when associated with an endometrioma, it poses a significant diagnostic challenge because of its close resemblance to ovarian malignancy [6]. In this report, we present a case of endometrioma associated with hemorrhagic ascites, pleural effusion, and elevated tumor markers, collectively mimicking ovarian neoplasia.

## Case presentation

**Figure 1:**
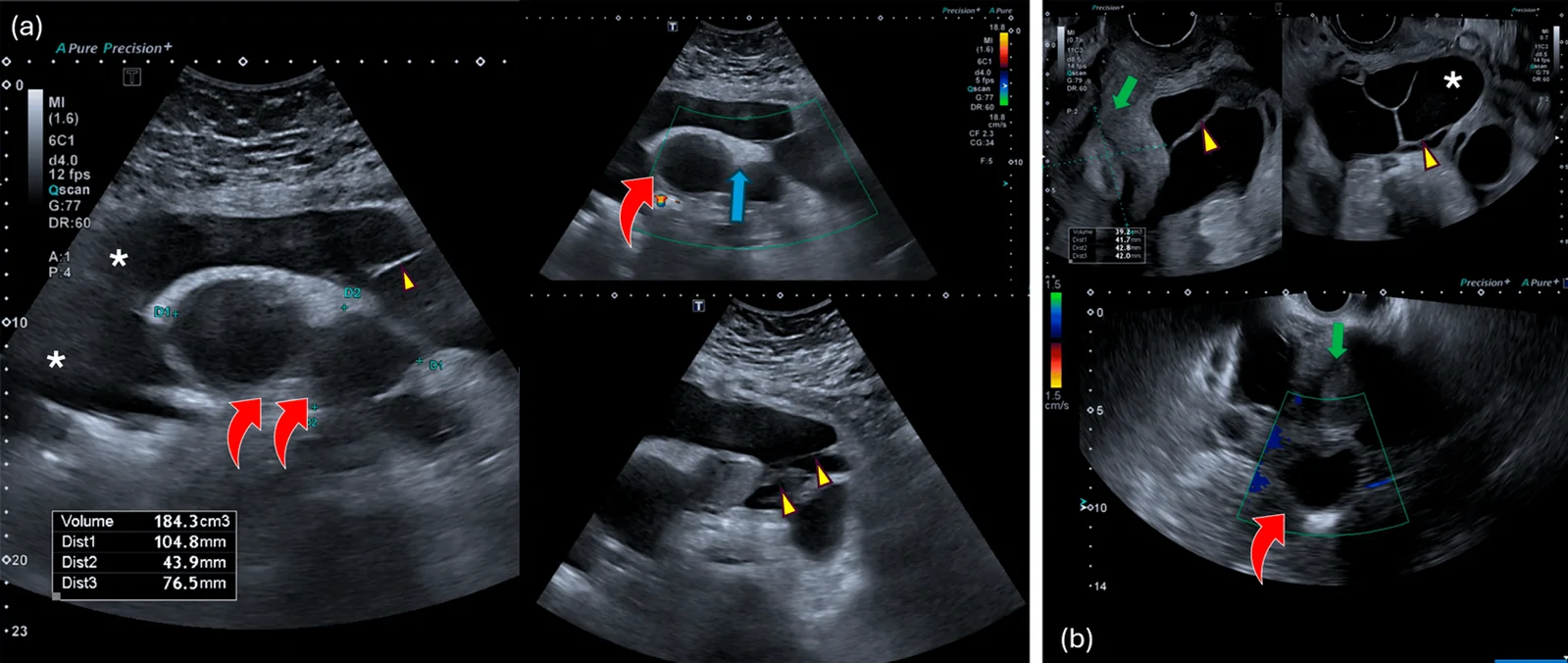
(a) Transabdominal gynecological ultrasound and (b) transvaginal ultrasound Lobulated collection vs. cystic adnexal lesion (red arrows). No flow on color Doppler (blue arrow). Abundant ascites with a non-free appearance, multiple septa (yellow arrowhead) in pelvic recesses, and partially particulate content (asterisks). The uterus appears normal (green arrow).

**Figure 2:**
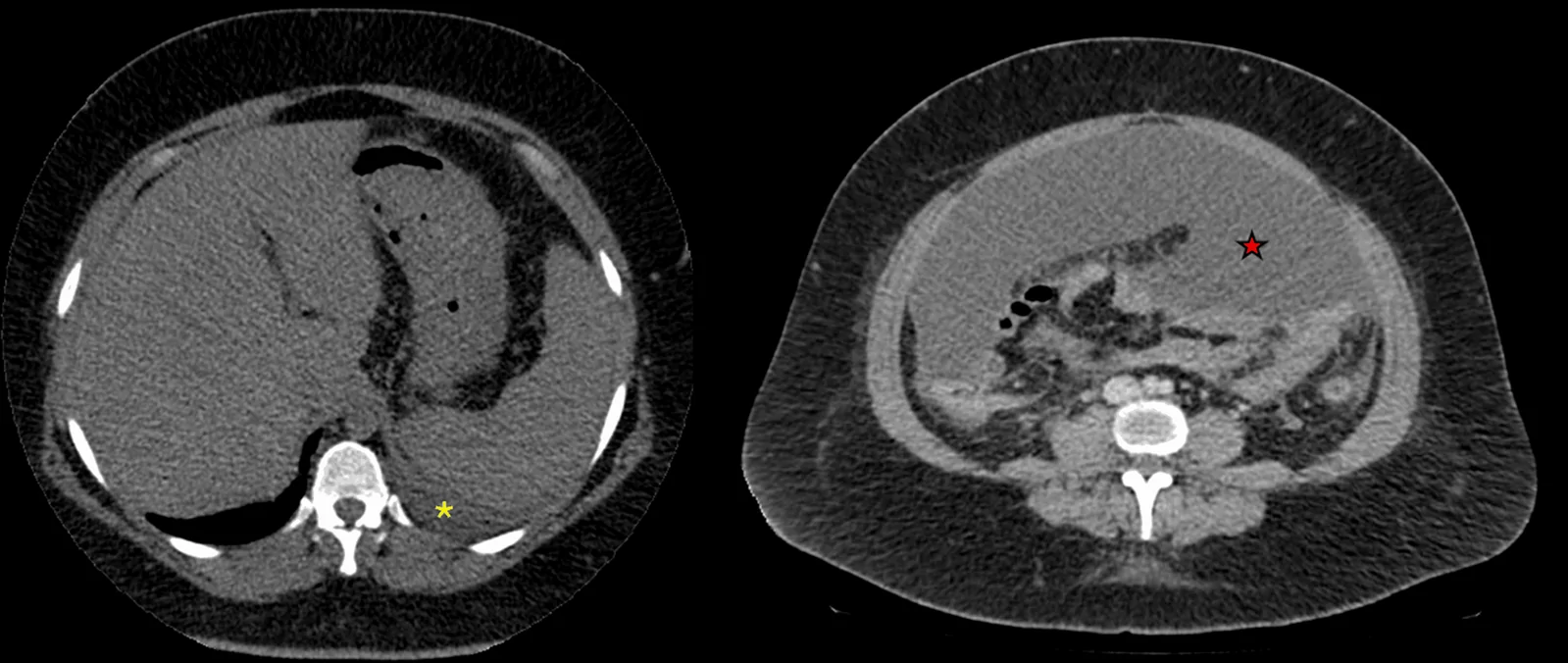
Pre- and post-contrast abdominal CT scan (axial view) Small left pleural effusion (asterisk) and ascites with atypical distribution in the inframesocolic intraperitoneal compartment (red star). CT: computed tomography

**Figure 3:**
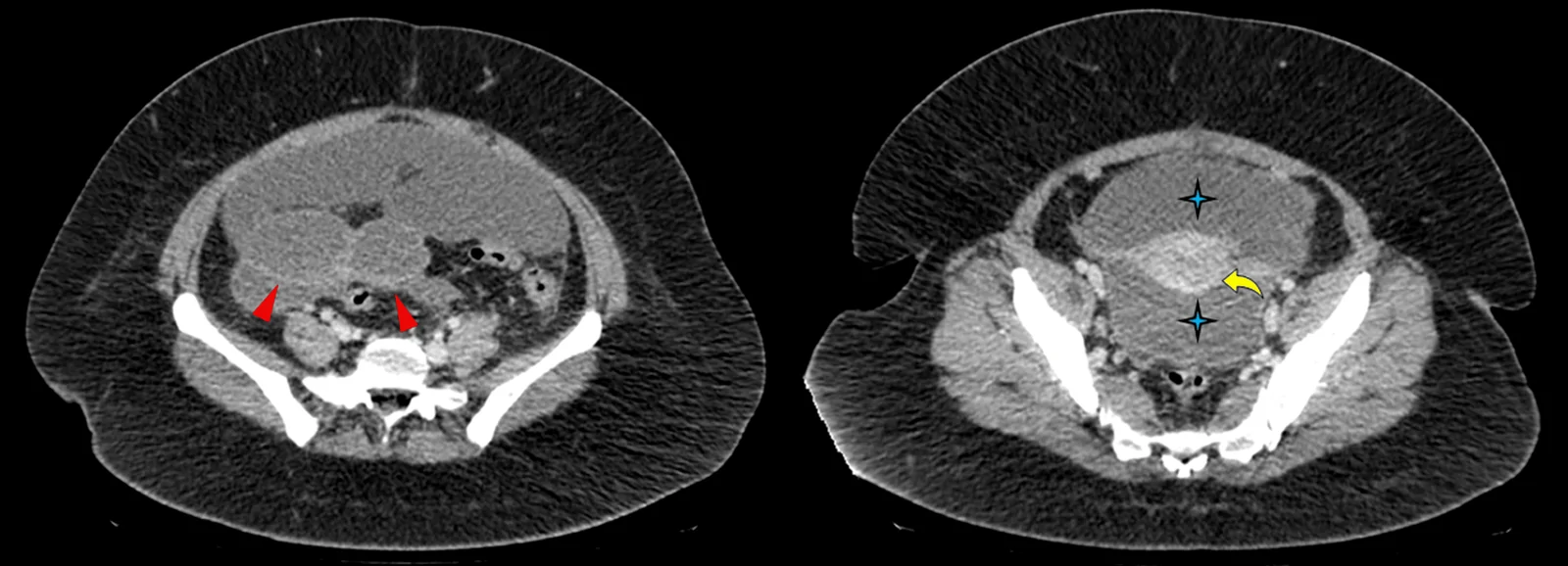
Abdominal CT scan (axial view) Right adnexal lesion with a cystic appearance, well-defined walls (red arrowheads) and fluid in the pelvic cavity (blue stars); uterus appears normal (yellow curved arrow). CT: computed tomography

We present the case of a 32-year-old female patient with a history of Class III obesity, salpingectomy (side unknown), and cesarean section, who presented with subacute abdominal pain in the right iliac fossa without peritoneal irritation, a negative pregnancy test, and no leukocytosis. At admission, abdominal ultrasonography revealed a suspected right iliac fossa collection associated with septated ascites. Transvaginal ultrasonography was of limited diagnostic value because of the location of the lesion (Figure 1); abdominal computed tomography (CT) demonstrated abundant ascites with atypical distribution, minimal left pleural effusion (Figure 2), and a right adnexal cystic lesion with associated pelvic free fluid (Figure 3). Paracentesis revealed abundant red blood cells.

**Figure 4:**
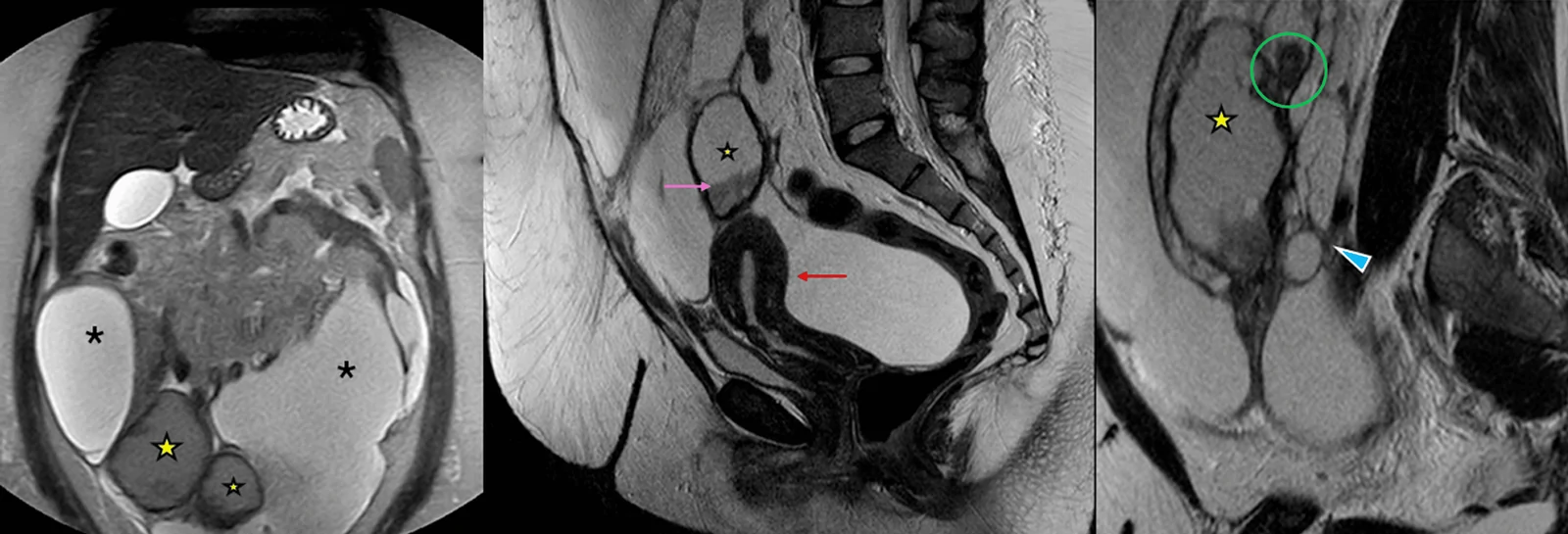
Abdomen and pelvis MRI (coronal and sagittal T2) The lesion shows high T2 signal intensity with a shading sign (yellow stars) and thick walls. Ascites is present in the inframesocolic compartment (asterisks). A fluid-fluid level is also observed (pink arrow). The uterus is unremarkable (red arrow). The bilobed lesion is adjacent to the anterosuperior margin of the right ovary (blue arrowhead) and the distal ileum (green circle). MRI: magnetic resonance imaging

**Figure 5:**
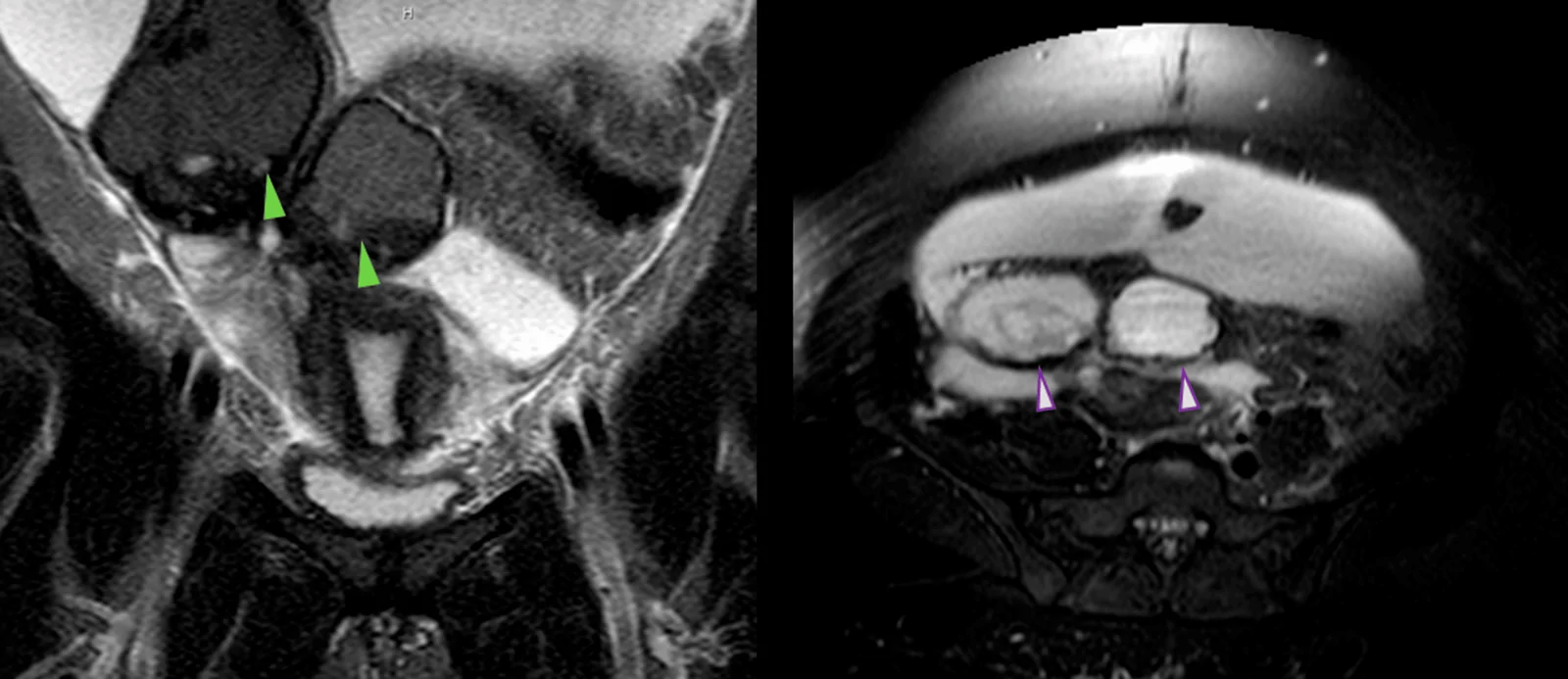
Pelvis MRI (coronal STIR and axial T2-Fatsat) The lesion shows a signal drop on inversion-recovery sequences (green arrowheads), a finding that can be mistakenly assumed to represent fat, but it remains high signal on T2 fat-saturated (T2-FS) images (pink arrowheads). The STIR sequence suppresses the signal from tissues with short T1 times, including methemoglobin, as in this case. MRI: magnetic resonance imaging; STIR: short tau inversion recovery

**Figure 6:**
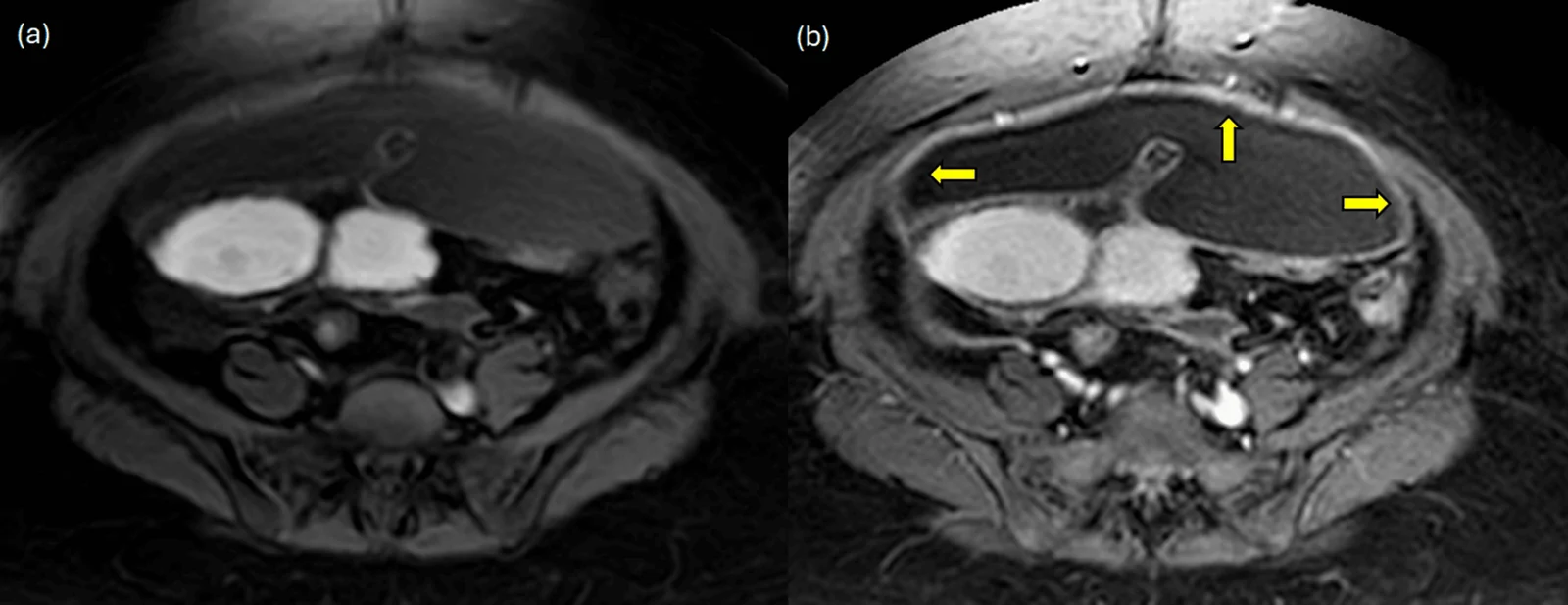
Pelvic MRI: (a) fat-suppressed T1-weighted and (b) contrast-enhanced T1-weighted sequences The lesion is intrinsically hyperintense on the pre-contrast T1 fat-saturated sequence due to its methemoglobin content, without enhancement of any internal components on the contrast-enhanced sequence. Note the enhancement of the parietal peritoneum due to chronic inflammation and irritation from blood products (yellow arrows). MRI: magnetic resonance imaging

**Figure 7:**
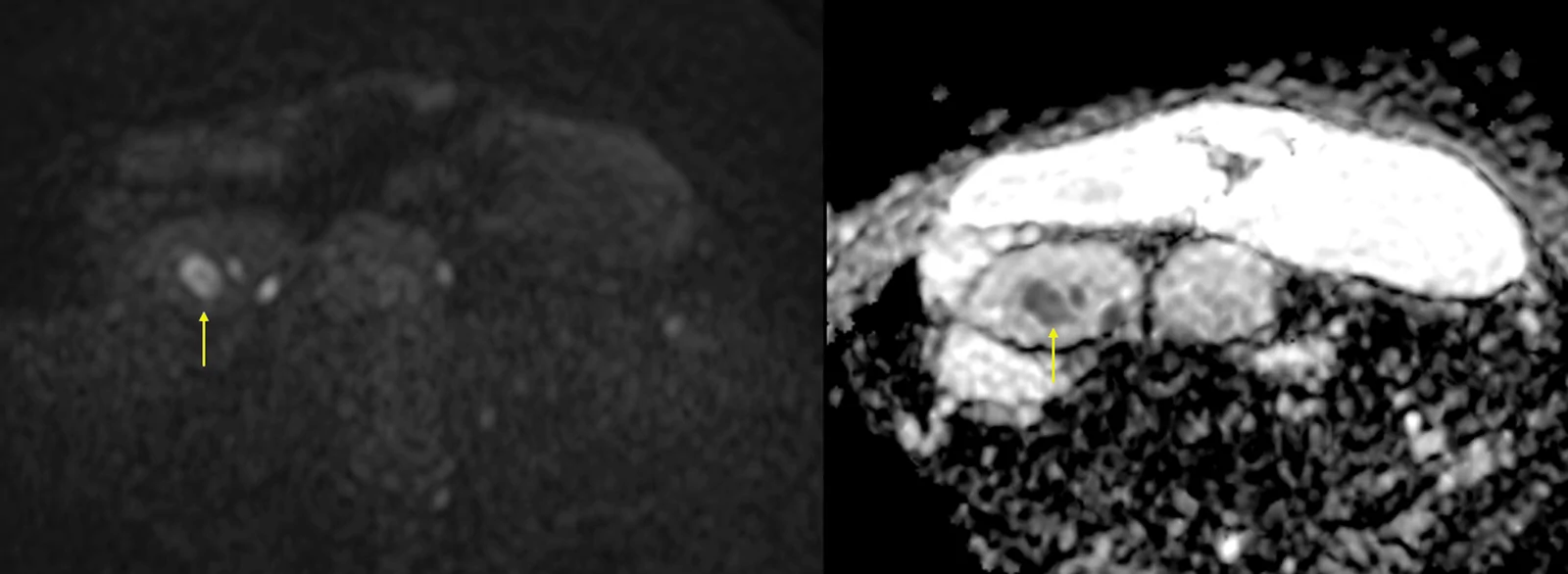
Pelvic MRI: DWI and ADC map There are foci of proton diffusion restriction in dependent areas of the lesions due to the presence of concentrated blood products (yellow arrows). MRI: magnetic resonance imaging; DWI: diffusion-weighted imaging; ADC: apparent diffusion coefficient

Abdominal and pelvic MRI showed a bilobed lesion with hemorrhagic content and thick walls in the upper aspect of the right ovary, in contact with the distal ileum (Figures 4-5). In addition, contrast enhancement of the peritoneum was noted (Figure 6), along with a few scattered foci of diffusion restriction in the dependent portions of the lesion (Figure 7), findings that together suggested endometriomas.

Tumor markers showed a significant elevation of cancer antigen 125 (CA-125) (385.5 U/mL) and cancer antigen 19-9 (CA 19-9) (630.6 U/mL); carcinoembryonic antigen (CEA) and alpha-fetoprotein were within normal limits (Table 1).

**Table 1:** Tumor marker levels High levels of CA-125 and CA-19-9 with reference ranges and units [6,7]. Carcinoembryonic antigen (CEA) and alpha-fetoprotein (AFP) levels were within normal limits.

Tumor Biomarker	Result	Reference Range	Units
Cancer Antigen 125 (CA-125)	385.5	0-35	U/mL
Cancer Antigen 19-9 (CA 19-9)	630.6	0-37	U/mL

Given the absence of a history of chronic pelvic pain or infertility, together with the tumor marker profile, a primary gastrointestinal malignancy was considered in the differential diagnosis. Upper gastrointestinal endoscopy and total colonoscopy were therefore performed to exclude a primary gastrointestinal neoplasm, with unremarkable findings (not shown).

The patient underwent an exploratory laparotomy with resection of a retroperitoneal tumor for suspected ovarian malignancy, followed by total hysterectomy, left salpingectomy, pelvic lymphadenectomy, omentectomy, and ablation of deep infiltrating endometriosis. Intraoperative findings (not shown) included severe pelvic adhesions, serosanguineous ascites with a characteristic "chocolate-like" appearance, adnexal masses adherent to the right colon, and enlarged iliac and obturator lymph nodes. The final histopathological report revealed superficial and deep infiltrating endometriosis without evidence of malignant transformation. The postoperative course was uneventful, and the patient was discharged in good condition.

## Discussion

Endometriomas, commonly referred to as "chocolate cysts," are lesions classically described on ultrasound as unilocular cysts without Doppler flow, exhibiting hypoechoic echotexture with a "ground-glass" appearance [8]. On MRI, they appear as rounded or oval lesions that are hyperintense on T1-weighted sequences and demonstrate low signal intensity on T2-weighted sequences, a finding known as the "shading sign," along with fluid-fluid levels [9]. Diffusion restriction is variable depending on the stage of hemoglobin degradation, while the presence of a solid enhancing component suggests malignant transformation [10].

In our case, transvaginal ultrasonographic evaluation was limited by the location of the lesion, despite the generally high diagnostic performance of ultrasound for endometrioma detection [5]. The transabdominal approach presented a broad differential diagnosis because of the lobulated appearance, thick walls, and concomitant ascites, ranging from hematosalpinx to inflammatory or infectious collections and low-grade ovarian neoplasms.

The presence of septated ascites with peritoneal enhancement and left pleural effusion are findings typically associated with malignant neoplastic processes. These findings can even be confused with multiloculated malignant cystic lesions, especially given the elevated tumor markers, which suggest a neoplastic etiology of ovarian or gastrointestinal origin. 

Hemorrhagic ascites represents an exceptionally rare manifestation of endometriosis; a systematic review identified 84 cases of histologically proven endometriosis-related hemorrhagic ascites between 1957 and 2018, of which 36 were associated with endometriomas [11]. This entity poses a major diagnostic challenge, as the triad of ascites, adnexal masses, and elevated tumor markers often mimics ovarian malignancy, directly impacting management. Multiple cases have been described in which the difficulty of establishing this distinction preoperatively has had significant implications for patient management and outcomes [11-16].

Tumor markers such as CA-125 and CA 19-9, when elevated, can occasionally misdirect the diagnosis toward malignancy; however, it must be emphasized that these are markers of low specificity [17]. While CA-125 is traditionally associated with non-mucinous epithelial ovarian carcinoma, it also increases during physiological conditions such as pregnancy and menstruation, as well as in other benign inflammatory conditions, including endometriosis, uterine leiomyomas, liver disease, renal disease, and heart failure [7]. Similarly, CA 19-9 has proven useful in detecting pancreatic and gastrointestinal cancers; however, its elevation has also been demonstrated in ovarian cancer, endometriosis, and mature cystic teratomas [6].

In our case, the elevation of these tumor markers heightened suspicion for malignancy and prompted an active search for gastrointestinal tumors, which yielded no findings. The interpretation of elevated tumor markers therefore requires careful consideration of both benign and malignant possibilities.

Imaging findings can be equivocal in reaching a definitive preoperative diagnosis. Both radiologists and clinicians must be aware of this limitation and of the broad spectrum of manifestations of benign pathologies that can simulate malignancy.

Some case reports highlight the utility of intraoperative histopathological analysis through frozen section biopsy [9,12], and several studies support this approach. A Cochrane review of 38 retrospective studies involving 11,181 patients found that frozen section biopsy had a concordance rate with paraffin-processed biopsies of 94% for benign tumors and 99% for malignant tumors [18].

## Conclusions

This report presents the case of a young woman with an adnexal mass, elevated tumor markers, and hemorrhagic ascites. This triad often mimics ovarian neoplasms, which impacts preoperative management. Multiple cases have been described in which the difficulty in distinguishing between the two has had repercussions on patient outcomes. Tumor marker levels may be misleading in inflammatory conditions. Therefore, it is essential to include endometriosis in the differential diagnosis of these atypical cases, both clinically and radiologically, even in the absence of prior pelvic pain or infertility.

This report is subject to the inherent limitations of a single-case design, which restricts the generalizability of its findings; further multicenter studies or case series could corroborate the frequency of this association. Additionally, long-term follow-up was limited. Imaging surveillance is recommended to rule out recurrence or the coexistence of an undetected malignancy.
